# Sustainable Cellulose-Based Gels: Synthesis, Chemical Modification, and Biomedical Application

**DOI:** 10.3390/gels12070648

**Published:** 2026-07-20

**Authors:** Bogdan-Marian Tofanica, Elena Ungureanu

**Affiliations:** “Ion Ionescu de la Brad” Iasi University of Life Sciences, 3 Mihail Sadoveanu Alley, 700490 Iasi, Romania; bogdan.tofanica@iuls.ro

**Keywords:** cellulose hydrogels, drug delivery systems (DDS), sustainable biomaterials, bio-based excipients

## Abstract

The growing demand for sustainable, biocompatible, and non-toxic biomaterials has driven significant advancements in biobased gels for biomedical applications. Among these, cellulose—the most abundant renewable biopolymer—presents an ideal platform due to its inherent hydrophilicity, structural tunability, and biodegradability. This review reports the recent advancements in the processing and engineering of cellulose-based hydrogels for drug delivery systems. We systematically explore the primary synthesis routes, including physical, chemical, and hybrid cross-linking strategies. Special emphasis is placed on chemical modifications (e.g., sulfation, carboxylation, etherification, and polymer grafting) that allow precise tuning of the gel’s mechanical strength, swelling kinetics, and stimuli-responsiveness (such as pH, temperature, or enzyme sensitivity). Furthermore, the review highlights essential characterization techniques—spanning structural, morphological, and rheological evaluations—used to relate cross-link density to the water-holding capacity and network homogeneity. By leveraging their highly hydrated and porous 3D architectures, these modified cellulosic networks demonstrate exceptional efficiency in drug loading, controlled release, and targeted localized therapy. Finally, we discuss current challenges, including industrial scalability and mechanical stability, and provide future perspectives on integrating nanoparticles and bioactive moieties to develop “smart” drug-eluting matrices and wound care dressings. Ultimately, this review underscores the immense potential of cellulose-based gels in advancing both clinical outcomes and circular economy goals.

## 1. Introduction

Hydrogels are three-dimensional, cross-linked polymeric networks defined by their ability to absorb and retain a significant fraction of water while remaining structurally intact and water-insoluble [1]. Because of their exceptional hydration, transparency, permeability, and tissue-like flexibility, hydrogels possess a biomimetic architecture that makes them very attractive for biomedical interventions [2]. They are now foundational materials in applications ranging from moist wound dressings and tissue engineering scaffolds to localized drug delivery systems (DDS) and soft ophthalmic devices (such as contact and intraocular lenses) [3].

Historically, the biomedical and superabsorbent fields have relied heavily on synthetic, petroleum-derived polymers—such as poly(acrylic acid) [4], polyacrylamide [5], and poly(2-hydroxyethyl methacrylate) (PHEMA) [6]. While synthetic hydrogels offer reproducible and tunable mechanical properties, they suffer from critical biological and environmental limitations. Many synthetic networks are biologically inert, non-biodegradable, and carry the risk of releasing toxic unreacted monomers or degradation byproducts (such as acrylamide) that can trigger local inflammation and cytotoxicity [7]. As clinical material science increasingly embraces the principles of a “circular economy,” there is an urgent imperative to replace these non-degradable, petroleum-derived super-absorbents with sustainable, non-toxic, and inherently biocompatible alternatives [8].

Cellulose has emerged as the premier candidate to bridge this gap. As the most abundant renewable biopolymer on Earth, cellulose forms the basis of sustainable, bio-based gels. Structurally, cellulose is a linear homopolysaccharide composed of repetitive β-D-glucopyranose units linked by β-(1→4)-glycosidic bonds. Each repeating glucose unit contains three reactive hydroxyl groups, making the individual chains inherently hydrophilic [9].

However, native cellulose presents distinct processing challenges. These abundant hydroxyl groups participate in extensive intra- and intermolecular hydrogen bonding, locking the polymer chains into a dense, highly ordered I-type crystal structure [10]. This robust crystallinity renders native cellulose completely insoluble in water and most standard organic solvents. To convert raw cellulose into a highly porous, water-swollen hydrogel that can match or exceed the tensile strength of synthetic equivalents, its crystalline domains must be disrupted. In practice, this is achieved by either dissolving the polymer in specialized non-derivatizing solvent systems (such as LiCl/N,N-dimethylacetamide or NaOH/urea aqueous solutions) to allow homogeneous gelation, or by chemically modifying the polymer backbone to introduce reactive or charged groups that facilitate water uptake and cross-linking [11].

The primary objective of this review is to systematically examine recent advancements in the processing, engineering, and biomedical application of cellulose-based hydrogels. We will trace the lifecycle of these biomaterials across the following domains:

- Synthesis and Cross-Linking Strategies: An exploration of how cellulose networks are constructed, contrasting physical methods (e.g., freeze–thaw cycles and ionic gelation) with robust chemical mechanisms (e.g., esterification via non-toxic cross-linkers like citric acid) and hybrid methodologies.
- Chemical Modification Strategies: A detailed analysis of how targeted chemical alterations—specifically sulfation, carboxylation, etherification, and polymer grafting—impart bioactivity, alter swelling kinetics, and enable precise structural tuning.
- Characterization Techniques: A review of the critical analytical methods (spanning FTIR, XRD, SEM, thermal analysis, and dynamic rheology) used to establish structure–property relationships and quantify network architecture.
- Biomedical Applications and Future Perspectives: An overview of how these hydrated, tunable networks are currently deployed in drug delivery, wound care, and tissue engineering. Finally, we address current translational bottlenecks—such as the trade-off between mechanical strength and porosity—and highlight future pathways for integrating smart, stimuli-responsive elements into fully biobased matrices.

Despite the vast body of literature on biopolymer hydrogels, a critical gap remains between the availability of cellulose as a natural resource, its chemical processing, and its successful biomedical application. A comprehensive and systematic evaluation of cellulose-based hydrogels across these interconnected domains is essential, as material availability, synthesis, modification, and application collectively constitute a continuous molecular engineering pathway. Native cellulose exhibits high crystallinity, strong intermolecular hydrogen bonding, and limited biological activity, which restrict its direct use in biomedical settings. Consequently, its clinical translation relies on effective strategies to disrupt its recalcitrant, hydrogen-bonding network and introduce functional chemical groups capable of meeting the stringent requirements of physiological environments.

Rather than treating these processes as isolated steps, a comprehensive evaluation of their interrelationship is essential to move the field away from empirical, trial-and-error approaches toward rational material design. By directly correlating molecular-level modifications (such as charge density or polymer grafting) to macro-scale clinical outcomes (such as controlled drug release and tissue integration), this review establishes a clear roadmap for researchers. This systematic approach is critical for overcoming the translational bottlenecks of mechanical instability and sterilization, ultimately transitioning cellulose hydrogels from laboratory-scale biomaterials into scalable, clinically viable, and environmentally sustainable medical application.

Ultimately, this review underscores how the strategic engineering of cellulose hydrogels can simultaneously fulfill the stringent demands of modern clinical therapies and global circular economy goals.

## 2. Synthesis and Cross-Linking Strategies

**Figure 1:**
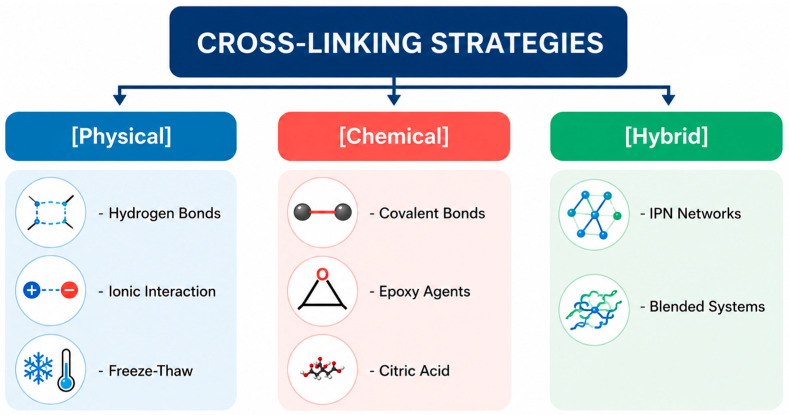
Classification of cross-linking strategies for cellulose-based hydrogels.

Before cross-linking can occur, native cellulose must often be transitioned from its highly crystalline, insoluble state into a processable form. A common synthesis route relies on direct dissolution using specialized non-derivatizing solvent systems, such as lithium chloride/N,N-dimethylacetamide (LiCl/DMAc) or aqueous NaOH/urea [12]. By activating the cellulose and fully dissolving it to form a homogeneous polymer solution, the material can be cast into molds. Gelation is then induced—often by introducing a non-solvent to trigger phase inversion or by allowing the solution to stand. The resulting hydrated cellulose networks can be washed, dried, and rehydrated to produce strong, transparent hydrogels [13]. To stabilize these 3D networks and tailor their physical properties, the cellulose chains are subjected to physical, chemical, or hybrid cross-linking strategies, as summarized in Figure 1. These routes range from reversible physical associations, which prioritize biocompatibility and simplicity, to robust covalent strategies designed for long-term structural stability. Furthermore, hybrid architectures have emerged as an effective means to engineer multi-functional materials that synergize the benefits of both chemical and physical networks.

### 2.1. Physical Cross-Linking

Physical cross-linking relies on reversible, non-covalent interactions to form hydrogel networks, offering the distinct advantage of avoiding potentially toxic chemical cross-linking agents, which renders these gels particularly desirable for sensitive biomedical applications [14].

One prominent method is freeze–thaw cycling, in which cellulose solutions or dispersions are subjected to repeated thermal transitions. The freezing phase induces microphase separation, forcing water into ice crystals and crowding the polymer chains into highly concentrated domains [15]. This close proximity promotes spontaneous crystallite formation and extensive chain entanglements; upon thawing, these physical junction zones remain stable, effectively locking the cellulose into a highly porous, physically cross-linked network.

Alternatively, ionic gelation utilizes the electrostatic behavior of cellulose derivatives bearing charged functional groups to achieve rapid gelation [16]. Carboxymethylcellulose (CMC), for instance, serves as an anionic precursor that readily forms hydrogels upon the addition of multivalent cations such as Al^3+^, or Fe^3+^ [17]. These ions function as cross-linking bridges by coordinating with negatively charged carboxylate (–COO^−^) groups on adjacent polymer chains. Recent advancements have significantly expanded the utility of this mechanism, as evidenced by the development of multifunctional materials; the integration of Al^3+^ into a transparent CMC solution, when combined with a fluorescent citrate-based moiety, has been shown to produce luminescent, self-healing hydrogels where metal ions provide dynamic, reversible cross-links while the citrate component confers additional biofunctionality [18].

### 2.2. Chemical Cross-Linking

To achieve superior mechanical stability, high fluid uptake, and precise control over degradation rates, chemical cross-linking is employed to introduce permanent covalent or exceptionally strong ionic bonds between cellulose chains [19]. A classic and environmentally benign method for this purpose is esterification via polycarboxylic acids, such as citric acid. When water-soluble CMC is mixed with citric acid and subjected to thermal curing—typically at temperatures ranging from 70 to 110 °C—the heat facilitates a dehydration reaction [20]. During this process, the citric acid forms cyclic anhydride intermediates that subsequently react with the hydroxyl groups of the cellulose backbone to establish robust ester linkages. This thermal cross-linking effectively converts a water-soluble polymer blend into a superabsorbent, water-insoluble network characterized by a significantly enhanced elastic modulus [21].

Beyond esterification, epoxy and radical cross-linking strategies offer additional pathways for network stabilization. Reagents such as epichlorohydrin or poly-epoxy compounds are frequently utilized to covalently bridge the hydroxyl groups of cellulose under alkaline conditions [22]. Alternatively, high-energy irradiation, including gamma rays electron-beam radiation. and UV radiation, can be applied to cellulose derivative solutions to generate free radicals directly along the polymer backbone. These macroradicals recombine to form a chemically cross-linked network, providing an effective synthetic route that is entirely free of external chemical initiators or traditional cross-linking agents [23].

Finally, the strong ionic pairing of functionalized cellulose allows for highly specialized network formation. By introducing charged groups prior to gelation, researchers can manipulate the self-assembly of the polymer. For example, sulfated cellulose nanofibers (CNF), which feature densely packed anionic –OSO_3_H groups, can be cross-linked using polyamines or chitosan [24]. In this system, the anionic sulfate esters and the cationic amino groups of the cross-linker engage in strong electrostatic interactions to yield a robust, three-dimensional ion-pair network. By fine-tuning the stoichiometric ratio of sulfate groups to amine cross-linkers, researchers can precisely control both the cross-link density and the resulting swelling kinetics of the hydrogel.

### 2.3. Hybrid Approaches

To overcome the inherent limitations of employing a single cross-linking modality—such as the physical brittleness often associated with heavily cross-linked chemical gels or the tendency of purely physical gels to dissolve prematurely in physiological fluids—hybrid strategies combine both approaches to optimize material performance [25]. A standard hybrid protocol involves inducing initial physical gelation, such as through freeze–thaw cycling, followed by a subsequent chemical fixation step to permanently lock the 3D network in place [26]. Furthermore, semi-interpenetrating polymer networks (semi-IPNs) or full IPNs are commonly engineered by blending or polymerizing a secondary synthetic network, including polyacrylamide or poly(ethylene glycol) (PEG), directly within the primary cellulose scaffold [27]. This approach effectively synergizes the tunable responsiveness and mechanical robustness of synthetic polymers with the inherent biocompatibility and sustainability of cellulose.

Advanced processing techniques further exploit these hybrid designs to achieve exceptional mechanical performance that surpasses traditional gel structures. For example, recent developments described in patent literature include methodologies where cellulose is extruded through syringe arrays into highly aligned fibers. By systematically stacking these oriented layers and subjecting them to subsequent cross-linking, researchers can produce laminated, anisotropic hydrogels that demonstrate vastly superior tensile strength, successfully mimicking the complex load-bearing capabilities found in natural biological tissues [28].

## 3. Chemical Modification Strategies

Chemical modification of the cellulose backbone is fundamental to tailoring hydrogel functionality. By altering the inherent chemical structure of the polymer, researchers can achieve precise tuning of the gel’s mechanical strength, swelling kinetics, and stimuli-responsiveness to environmental triggers such as pH, ionic strength, or temperature [29]. In all modification strategies, the degree of substitution is a principal parameter that is carefully controlled to balance water uptake capacity with the maintenance of structural integrity [30].

**Figure 2:**
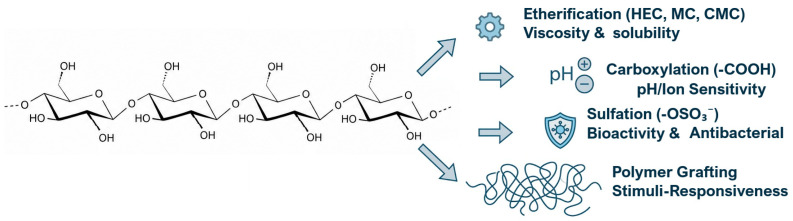
Schematic representation of common chemical modification strategies for cellulose: Etherification, Carboxylation/Carboxymethylation, Sulfation, and Polymer grafting.

The versatility of cellulose as a biomaterial scaffold is largely derived from the chemical reactivity of its hydroxyl groups. By selectively substituting these moieties, the physicochemical properties of the cellulosic network can be precisely tailored to meet the demands of specific biomedical environments [31]. As illustrated in Figure 2, chemical modification allows for the introduction of distinct functional handles—such as anionic charges or stimuli-responsive synthetic grafts—which dictate the gel’s interaction with the biological milieu.

These modifications act as the primary design levers for balancing material performance. For instance, the transition from raw cellulose to a functionalized derivative allows for the decoupling of mechanical stiffness from water-holding capacity. While carboxylation effectively enhances ion-binding and swelling in physiological buffers, sulfation offers a pathway to combine structural integrity with antibacterial bioactivity. Furthermore, grafting synthetic polymer brushes creates a ‘smart’ interface, where the hydrogel can undergo conformational changes in response to external stimuli. By modulating the degree of substitution, researchers can bridge the gap between a simple passive scaffold and a multifunctional, responsive drug-eluting matrix.

### 3.1. Etherification (Synthesis of CMC, MC, HEC)

Etherification, including alkylation, introduces bulky side-groups to the cellulose backbone to synthesize derivatives such as hydroxyethyl cellulose (HEC) and methylcellulose (MC) [32]. These modifications effectively disrupt the dense native hydrogen-bonding network, thereby improving aqueous solubility and significantly altering the polymer’s thermal properties. Etherified celluloses can form hydrogels with unique rheological profiles; for instance, modified hydroxyethyl cellulose has been shown to form gels with enhanced UV-curing capabilities [28]. State-of-the-art literature also highlights advanced strategies, such as one-pot etherification combined with self-crosslinking via a mild hydroxylyne click reaction, which yields cellulose hydrogels that rapidly stiffen under UV exposure [33]. Such ether-based modifications not only improve the baseline mechanical strength of the matrix but also introduce reactive double bonds that facilitate further chemical grafting applications.

### 3.2. Carboxylation and Sulfation (Altering Charge Density and Swelling Dynamics)

Carboxylation and sulfation are essential strategies for fundamentally altering the charge density and swelling dynamics of cellulose hydrogels [34]. Carboxylation, notably through the synthesis of CMC, introduces anionic –COOH or –COO^−^ groups. This modification renders the previously insoluble cellulose highly water-soluble and imparts significant pH and ion sensitivity to the resulting network [35]. Furthermore, these carboxylate groups enhance drug-binding capacities through ion pairing and facilitate pH-responsive swelling. As previously established, CMC chains can be readily cross-linked via multivalent cations or covalently bonded via esterification with polycarboxylic acids (such as citric or succinic acid) under heat to yield gels with exceptionally high absorption capacities [36].

Similarly, treating cellulose with sulfating agents, such as sulfuric or sulfamic acid, incorporates densely packed anionic –OSO_3_^−^ groups. These negative charges enable robust ionic cross-linking with amine-containing cross-linkers [37,38]. Sulfated nanocellulose represents a particularly novel functional material: it retains the native I-crystallinity of cellulose and remains water-insoluble, yet it becomes exceptionally hydrophilic [39]. This sulfated cellulose nanofiber (CNF) network can be completely dried and subsequently redispersed, providing good handleability for manufacturing [40]. Literature emphasizes that sulfated CNF hydrogels are suitable for applications requiring intense moisturizing, water retention, antibacterial and antiviral properties, advanced wound care, and localized drug delivery [39]. Ultimately, sulfation introduces bioactive moieties that can act as physiological heparin mimetics while providing a strong capability for ionic cross-linking. Crucially, the functional success of these gels depends heavily on the degree of modification [41]. For example, an optimal degree of sulfation—typically ranging from 0.05 to 1.0 sulfate groups per glucose unit, with an ideal target of 0.15 to 0.6—is rigorously specified to perfectly balance maximal water uptake with the preservation of crystalline integrity [42].

### 3.3. Polymer Grafting (Enabling Stimuli-Responsiveness)

To further expand the functionality of cellulose gels, polymer grafting and composite formation are frequently employed to engineer hybrid networks. Cellulose can be graft-copolymerized with various synthetic monomers to synergize the inherent biocompatibility of the natural biopolymer with the tunable physical properties of synthetic systems. In practice, hydrogels often comprise a cellulose scaffold blended with or covalently bonded to secondary polymers such as hydroxyethyl methacrylate (HEMA), poly(2-hydroxyethyl methacrylate) (PHEMA), polyacrylamide, poly(ethylene glycol) (PEG), poly(vinyl alcohol) (PVA), and poly(vinylpyrrolidone) (PVP) [43]. Grafting these synthetic polymers onto cellulose chains—typically via radical polymerization mechanisms—is an effective method for yielding true stimuli-responsive materials. A prominent example is the grafting of poly(N-isopropylacrylamide) (poly(NIPAAm)) onto a cellulose backbone, which imparts distinct, reversible temperature-responsive swelling behaviors to the hydrogel [44]. By strategically building synthetic polymer networks around a chemically modified cellulose scaffold, researchers can engineer “smart” biomaterials with customized environmental responsiveness and specific mechanical profiles.

In all cases, the degree of substitution matters. For instance, the CNF sulfate literature specifies an optimal degree of sulfation (∼0.05–1.0 sulfate groups per glucose unit, ideally 0.15–0.6) to balance water uptake with crystalline integrity [45]. Similarly, carboxymethylation levels are controlled to maintain gel strength. These modifications allow “precise tuning of the gel’s mechanical strength, swelling kinetics, and stimuli-responsiveness” (pH/ion, temperature, etc.) as highlighted in the literature [46].

## 4. Characterization Techniques

**Figure 3:**
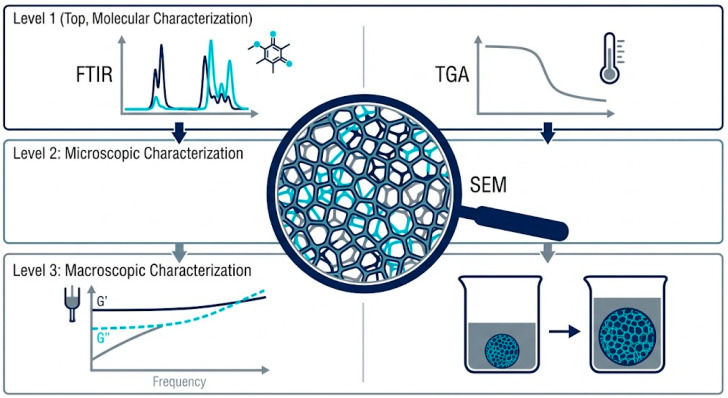
Multi-scale characterization workflow for cellulose-based hydrogels. The analytical strategy spans: (Level 1) Molecular and thermal analysis (FTIR, TGA, XRD) to confirm chemical identity and thermal stability; (Level 2) Microscopic evaluation (SEM) to quantify 3D network porosity; and (Level 3) Macroscopic assessment (Rheology and Swelling kinetics) to determine mechanical viscoelasticity and water-uptake efficiency.

To establish the structure–property relationships required for clinical application, cellulose hydrogels are subjected to a rigorous, multi-scale analytical workflow. As summarized in Figure 3, this methodology transitions from the molecular and thermal analysis of the polymer backbone to the visualization of the internal porous microarchitecture, culminating in the macro-scale evaluation of viscoelasticity and fluid-uptake dynamics.

By integrating these datasets, a complete understanding of the hydrogel’s performance emerges. Spectroscopic and thermal data validate the precision of the chemical modifications, while SEM analysis reveals the morphological constraints governing mass transport. When synthesized with the corresponding rheological and swelling performance, these metrics provide a predictive model for the material’s behavior in vivo, ensuring that the scaffold maintains its structural integrity while simultaneously achieving the desired therapeutic release profiles.

### 4.1. Morphological and Structural Analysis

The fundamental confirmation of successful network formation and chemical modification begins with structural and spectroscopic analysis.

Fourier-transform infrared spectroscopy (FTIR) is routinely employed to verify the introduction of new functional groups—such as the distinct stretching vibrations of –OSO_3_^−^ in sulfated cellulose or –COO^−^ in carboxymethylated derivatives—and to confirm the establishment of covalent ester linkages during chemical cross-linking [47].

Complementing chemical analysis, X-ray diffraction (XRD) is utilized to assess the crystallographic transition of the material. While native cellulose exhibits a highly ordered I-type crystal structure, the dissolution, modification, and subsequent cross-linking processes typically reduce this crystallinity, yielding a predominantly amorphous hydrogel network [48].

In addition to structural crystallinity, evaluating the composition and thermal stability of the matrix is critical, which is achieved through thermal analysis techniques such as thermogravimetric analysis (TGA) and differential scanning calorimetry (DSC). TGA continuously tracks weight loss as the gel is heated; cellulose hydrogels characteristically exhibit an initial weight loss phase corresponding to the evaporation of free and tightly bound water, followed at higher temperatures by the thermal decomposition of the polymer backbone. Patent literature frequently relies on TGA curves of both dried and rehydrated gels to accurately quantify water retention capacities and confirm the thermal endurance of the matrix [49].

Finally, to visualize the resulting microarchitecture, scanning electron microscopy (SEM) is applied to freeze-dried samples (aerogels). SEM imaging reveals the internal three-dimensional porous network, allowing researchers to quantify pore size distribution and interconnectivity—typically ranging from tens to hundreds of micrometers. This porous morphology is a critical determinant of the gel’s permeability, directly influencing mass transport, localized drug diffusion rates, and the potential for cellular infiltration in tissue engineering constructs [50].

### 4.2. Rheological Evaluations

To translate structural characteristics into functional mechanical properties, dynamic rheological evaluations are performed. In the characterization of soft biomedical hydrogels, mechanical evaluation is strategically focused on rheological and viscoelastic behaviors (such as shear-thinning and storage/loss moduli dynamics) rather than destructive high-load structural mechanics. Viscoelasticity directly governs the clinical performance of these matrices, determining their suitability for minimally invasive injectability, mucoadhesive retention at target sites, and network stability under biological shear rates [41].

Rheology provides profound insights into the viscoelastic nature of these soft, heavily hydrated biomaterials. Through oscillatory stress testing, the fundamental hallmark of a stable hydrogel network is confirmed when the storage modulus (G′), which represents the elastic, solid-like energy storage of the material, significantly exceeds the loss modulus (G′′), denoting the viscous, fluid-like energy dissipation, across a broad frequency sweep. The magnitude of G′ directly correlates with the stiffness and cross-link density of the cellulosic matrix [51].

Furthermore, many modified cellulose hydrogels exhibit pronounced shear-thinning behavior. Under increasing shear rates, the physical entanglements or reversible cross-links within the network temporarily align or break, leading to a dynamic decrease in viscosity. This shear-thinning property is advantageous for clinical translation, as it dictates the material’s suitability for minimally invasive injectable drug delivery systems and its extrudability as a bioink in 3D bioprinting applications [52].

### 4.3. Swelling Kinetics and Network Homogeneity

The defining operational metric of any hydrogel is its capacity to absorb and retain water, which is quantitatively evaluated through swelling kinetics. Equilibrium water uptake is measured gravimetrically by immersing the desiccated polymer matrix in aqueous or physiological buffer solutions until mass constancy is achieved [53]. This swelling data is deeply intertwined with the material’s structural homogeneity and is frequently used to calculate the physical cross-link density of the network via thermodynamic models [54], such as the Flory-Rehner theory [55]. There is an inherent, inverse relationship between mechanical robustness and hydration: a densely cross-linked, highly homogeneous network heavily restricts the mobility of the cellulose chains, resulting in an elevated elastic modulus but a correspondingly lower maximum swelling ratio. Additionally, for stimuli-responsive functionalized celluloses, swelling kinetics are dynamically evaluated across varying pH levels, ionic strengths, or temperatures to map the gel’s responsive behavior [56]. Understanding these swelling profiles is vital, as the expansion of the network dictates the controlled release kinetics of loaded therapeutic agents and confirms the stability of the matrix against rapid physiological degradation [57].

By combining these techniques, researchers relate network architecture (crosslink density, porosity) to functional properties (water retention, strength, diffusion rates).

## 5. Biomedical Applications

Cellulose-based hydrogels are increasingly recognized as highly versatile platforms across a broad spectrum of biomedical applications. Their inherent biocompatibility, combined with exceptional hydration capabilities and a structurally tunable polymeric network, positions them as ideal materials for interfacing with sensitive biological tissues. The functional versatility of these hydrogels—spanning from passive structural scaffolds to active, responsive matrices—has driven significant translational research aimed at improving clinical outcomes [58].

**Figure 4:**
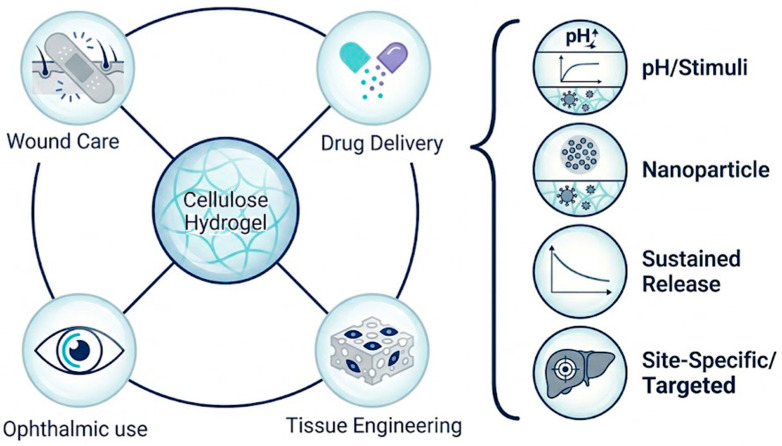
Biomedical applications of cellulose-based hydrogels, with a focus on Drug Delivery Systems (DDS). Cellulose functions as a versatile platform ((**left**): wound dressings, tissue engineering, and ophthalmic applications), with a major emphasis on DDS (**right**), where the 3D structure enables controlled release via: stimuli-responsive mechanisms (pH/temperature), nanoparticle integration, sustained release profiles, and site-specific targeting.

Due to its biocompatibility, non-toxic nature, and versatile functionalization capacity, cellulose has transcended its traditional role as a simple excipient [59]. As summarized in Figure 4, cellulose-based polymeric networks now serve as critical platforms across a diverse range of clinical applications, extending from the targeted delivery of therapeutic agents to complex tissue regeneration and next-generation ophthalmic devices.

The versatility of cellulose as a biomaterial is best illustrated by the breadth of its clinical applications. Figure 4 synthesizes this potential, highlighting the role of cellulose in wound care, tissue engineering, and ophthalmology. However, DDS represent the central pillar of current innovation. As detailed in the DDS branching pathways, modern design has moved beyond simple encapsulation, integrating advanced strategies for targeting, release control, and the utilization of nanotechnologies to transform the hydrogel into a ‘smart’ and very adaptable therapeutic system.

### 5.1. Drug Delivery Systems (DDS)

Cellulose hydrogels exhibit immense potential as sophisticated drug delivery systems, a domain that heavily exploits their highly porous, three-dimensional architecture and tunable physicochemical properties.

The clinical implementation of cellulose-based hydrogels represents a major advancement in pharmaceutical technology, shifting from the use of simple excipients to the development of sophisticated, active therapeutic carriers. This massive transition is fundamentally driven by the inherent biocompatibility, biodegradability, low toxicity, and multi-scale functionalization capacity of the cellulosic backbone [60,61,62]. Extensive reviews over the past decades confirm that cellulose and its derivatives—such as carboxymethyl cellulose (CMC), hydroxypropyl methylcellulose (HPMC), and hydroxyethyl cellulose (HEC)—are cornerstones for establishing sustained, controlled, and site-specific drug release formats, thereby overcoming classic challenges associated with systemic toxicity and poor drug loading [63,64,65,66,67,68].

#### 5.1.1. Fundamentals of Matrix Engineering and Release Kinetics

In classic hydrophilic matrices, the mechanism of drug delivery relies predominantly on the gelation behavior of the polymer upon contact with physiological fluids. For instance, cellulosic polymers like HPMC hydrate to form a gelatinous peripheral layer that dictates the diffusion of active molecules; release kinetics here are a complex interplay of Fickian diffusion, structural matrix erosion, drug dissolution, and the thermodynamic relaxation of the polymer chains [69,70]. The macro-mechanical and textural properties of these gels, such as hardness and compressibility, are heavily influenced by the polymer concentration and directly correlate with the formulation’s mucoadhesive strength and clinical spreadability [71]. To further enhance mechanical resilience, recent one-pot synthesis strategies have successfully co-polymerized CMC with polyacrylamide via cryo-UV irradiation, yielding highly porous, tough hydrogels that withstand cyclic compression while maintaining zero-order, sustained drug release profiles [72]. Additionally, incorporating macro-molecules such as carboxymethyl β-cyclodextrin into CMC matrices significantly bolsters compressive strength while maximizing the loading and controlled release of hydrophobic antibiotics [73]. The cross-linking of bacterial cellulose with gelatin using glutaraldehyde further establishes stable, interpenetrating networks exhibiting profound thermal resistance and high swelling ratios (400–600%) [74].

#### 5.1.2. High-Porosity Aerogels and Nanoscale Delivery Platforms

To drastically increase drug-loading capacity, processing techniques have evolved toward high-porosity architectures, notably aerogels and xerogels. These materials represent the dehydrated structural precursors or dried states of the described hydrogel networks. They allow for the study of the internal pore architecture in a fixed state, before their subsequent transition back into highly hydrated hydrogel matrices upon contact with physiological fluids. Processed via supercritical CO_2_ drying or low-vacuum evaporation from sustainable NaOH solutions, these lightweight materials achieve extraordinary specific surface areas (up to 680 m^2^/g) and porosities exceeding 90%, offering customizable release profiles governed by either pore-media diffusion or swollen-wall diffusion [75,76,77]. In parallel, nanotechnology has integrated cellulose nanocrystals (CNC) and nanofibrils into delivery systems to bypass the biological barriers that limit the absorption of Biopharmaceutics Classification System (BCS) Class II drugs [78,79,80]. For example, CNCs extracted from agricultural waste (rice husks) have been utilized as effective reinforcing agents within gelatin hydrogels, drastically improving the storage modulus and establishing pH-sensitive delivery carriers suited for theophylline administration [81].

#### 5.1.3. Smart, Stimuli-Responsive, and Interpenetrating Networks (IPNs)

The integration of stimuli-responsive features enables precise spatial and temporal control over therapeutic release. pH-responsive hydrogels are particularly prominent; utilizing layered double hydroxides (LDH) intercalated with ibuprofen inside a CMC matrix yields nanocomposite beads that protect the payload from harsh stomach acidity and ensure targeted intestinal release [82]. Similar targeted architectures include radiation-induced co-polymerization of CMC with acrylic acid for colon-specific release [83], as well as bacterial cellulose/acrylic acid hydrogels that exhibit highly tunable thermo- and pH-responsive swelling [84]. Cationic derivatives, such as Polyquaternium cross-linked with ethylene glycol diglycidyl ether, also demonstrate precise ion- and pH-triggered release kinetics driven by electrostatic interactions [85]. Advanced double-layer IPNs, utilizing a pH-sensitive alginate-CMC core protected by a chemically cross-linked synthetic outer layer, precisely regulate structural expansion and eliminate uncontrolled macromolecular diffusion [86]. Furthermore, incorporating biocompatible succinoglycan into CMC produces an IPN with an 8.5-fold improvement in compressive stress and efficient, pH-controlled release of 5-fluorouracil [87]. Cutting-edge designs have also realized photo-switchable and reduction-responsive systems, where azobenzene-grafted CMC utilizes host-guest complexation to self-heal and structurally degrade upon exposure to specific UV wavelengths [88]. Innovative complexation strategies also extend to cyclosophoraose/cellulose hydrogels, which act as efficient host-guest carriers for hydrophobic antibacterial drugs like galangin [89], and N-trimethyl chitosan-CMC systems cross-linked organically or via Cu(II) ions, achieving prolonged Fickian diffusion of ciprofloxacin [90].

#### 5.1.4. Route-Specific Applications: Oral, Ocular, and Topical Delivery

Route-specific constraints have driven the formulation of specialized cellulosic matrices. For oral gastroretentive delivery, hybrid gels of high-amylose starch and microcrystalline cellulose form low-density porous matrices capable of gastric floating and sustained osmotic-driven drug release for over 24 h [91,92]. In ophthalmic applications, rapid physiological drainage is circumvented using in-situ gelling systems combining Carbopol and methylcellulose; these liquid formulations rapidly undergo pseudoplastic phase transitions into stiff gels at ocular temperature and pH, drastically increasing the precorneal residence time of antimicrobials like pefloxacin mesylate [93,94,95].

For topical and mucosal applications, cellulose gels excel in maintaining moist wound environments and serving as anti-inflammatory barriers [96,97,98]. Advanced variations include emulgels formulated with cellulosic gums to deliver lipophilic molecules deeply into the epidermis [99], and non-aqueous ethyl cellulose systems utilizing propylene glycol dicaprylate that exhibit inverse thermoreversible gelation for specific dermal excepient applications [100]. In buccal delivery, HEC gels heavily loaded with solid lipid nanoparticles ensure ex vivo mucosal permeation and prolonged localized antimicrobial action for periodontal disease [101]. Similarly, lyophilized NaCMC wafers loaded with neomycin provide absorbent, antibacterial matrices for severe mucosal infections [102], while resveratrol-loaded cellulose aerogels mediate the P38 signaling pathway to profoundly reduce synovial inflammation in sports-related osteoarthritis [103].

#### 5.1.5. Advanced Therapeutics: Oncology and Immunotherapy

Perhaps the most critical translational boundary for cellulosic DDS is targeted oncology and transdermal tumor therapy [104]. Cellulose nanocrystals demonstrate profound efficacy as nanocarriers; for instance, CNCs loaded with 5-fluorouracil directly induce apoptosis and mitochondrial membrane degradation in colorectal cancer cell lines [105]. The targeting capability of these nanocarriers is further enhanced by incorporating Fe_3_O_4_ nanofillers coated with cross-linked chitosan, creating a magnetic CNC complex capable of deeply penetrating 3D tumor spheroids under external magnetic fields [106]. To address the formidable challenge of multidrug resistance in gastric cancer, thiolated CMC microgels have been formulated to co-deliver hydrophilic 5-FU alongside hydrophobic curcumin. This dual-drug microgel synergistically triggers reactive oxygen species (ROS)-mediated apoptosis, successfully overcoming single-agent resistance [107]. Finally, biocompatible bacterial cellulose (BC) has proven invaluable not only as a slow-release transdermal matrix for hydrophobic active ingredients encapsulated in block copolymer nanoparticles [108,109], but also as an implantable local delivery system for immune checkpoint blocking antibodies (e.g., anti-CTLA-4). By retaining these antibodies within the tumor microenvironment, BC prevents uncontrolled systemic spread, drastically mitigating the severe toxicities typically associated with systemic immunotherapy [110].

### 5.2. Advanced Wound Care and Dressings

In the realm of wound management, the high water content and absorbent nature of cellulose hydrogels make them exemplary materials for moist wound dressings [111]. Maintaining a properly hydrated microenvironment is clinically proven to accelerate epidermal regeneration, promote angiogenesis, and reduce scar formation [112]. The intrinsic porosity of the cross-linked cellulose network permits the continuous absorption and directional drainage of excess wound exudate, effectively preventing both tissue maceration and wound bed dehydration [113]. Furthermore, the optical transparency of many cellulose hydrogels allows clinicians to visually monitor the healing process without the need to painfully or disruptively remove the dressing [114]. Recent functional innovations have further elevated their utility; for example, sulfated cellulose nanofiber (CNF) hydrogels possess intrinsic antibacterial and antiviral characteristics. These bioactive dressings not only serve as a highly hydrated physical barrier but actively mitigate the risk of localized nosocomial infections [115].

### 5.3. Tissue Engineering Scaffolds

The morphological and physicochemical similarities between heavily hydrated, porous cellulose gels and the native extracellular matrix (ECM) render them reliable candidates for advanced tissue engineering scaffolds [116]. By employing specific processing techniques—such as freeze-drying to create structural aerogels or leveraging entangled nanofibrillated networks—researchers can fabricate biomimetic scaffolds tailored for specific tissue phenotypes, including complex bone and cartilage constructs [117]. Notably, sulfated CNF hydrogels have been explicitly highlighted in the literature as being uniquely suited for the development of artificial cartilage due to their dense hydration and mechanical resilience [118]. The long-term success of these cellular scaffolds relies on the precise tuning of network stiffness, mechanical elasticity, and controlled biodegradation rates, ensuring that the hydrogel can provide immediate structural support for cellular adhesion and proliferation before gradually resorbing as the de novo host tissue integrates [119].

### 5.4. Soft Ophthalmic Materials and Emerging Uses

Remarkably, the optical clarity and oxygen-independent durability of specific cellulose hydrogels—particularly those derived from bacterial cellulose—have spurred their investigation as next-generation soft ophthalmic materials, including contact and intraocular lenses [120]. Their high oxygen permeability and good hydrophilicity offer compelling long-term advantages over traditional synthetic counterparts (such as HEMA-based commercial lenses), particularly in maintaining ocular biocompatibility and reducing the incidence of dry-eye syndrome [121].

Beyond these primary biomedical fields, the profound versatility of cellulose extends into a myriad of emerging clinical and consumer applications. These bio-based gels are currently utilized as hydrating matrices in dermatological cosmetics (e.g., facial masks and moisturizing gels), structural, ion-conductive supports for biosensor electrodes, and functional coatings for drug-eluting vascular catheters. Looking forward, the integration of nanosensors, magnetic nanoparticles, or targeted growth factors directly into the cellulosic backbone is rapidly driving the development of “smart” biomedical devices [122]. Across all these diverse roles, the unifying clinical advantage remains the unique capacity of cellulose hydrogels to merge high hydration and rigorous biocompatibility with tunable, application-specific functionality [123].

### 5.5. Synthesis-to-Application Correlation: Comparative Design Matrices

To establish a rational roadmap for future biomaterial engineering, it is imperative to directly correlate molecular-level synthesis and chemical modification routes with their macroscopic, experimentally validated biological performance. This section compiles these multi-variable parameters into two comprehensive comparative design matrices, bridging the chemical engineering concepts detailed in the earlier chapters with the clinical outcomes discussed throughout Section 5.

In order to systematically evaluate how different network stabilization methods manifest as physical-chemical and biological performance metrics, a comprehensive comparison of physically, chemically, and hybrid cross-linked cellulose hydrogels is presented in Table 1.

The comparative data compiled in Table 1 underscores a fundamental design trade-off in network engineering. While physical cross-linking strategies [81,98] prioritize rapid gelation and maximum biosafety by avoiding potentially toxic chemical modifiers, they remain susceptible to mechanical failure and premature dissolution in physiological fluids. Conversely, chemical (covalent) networks [68,83,85] provide robust structural stability and high elastic moduli, yet they introduce significant translational hurdles related to the rigorous extraction of toxic chemical cross-linkers. Ultimately, hybrid and IPN strategies [72,86,87] successfully break this paradigm, offering a synergistic compromise where dynamic physical interactions and permanent covalent structures co-exist to yield tough, elastic, and biologically safe matrices suitable for demanding load-bearing applications.

In a similar manner, the specific chemical modification strategy applied to the cellulose backbone serves as the primary molecular handle for tailoring biological interactivity, aqueous solubility, and mass transport kinetics. Table 2 outlines the strategic advantages, disadvantages, crystalline structural impacts, and specific pharmaceutical applications of the three primary modification routes—etherification, carboxylation/sulfation, and polymer grafting.

As synthesized in Table 2, each chemical modification route addresses distinct clinical requirements. Etherification [93,94,101] is the preferred molecular strategy when solubility optimization and stable, non-ionic mucoadhesive barriers are required, making it effective for ocular and mucosal delivery. Carboxylation and sulfation [71,82,102], on the other hand, are indispensable for introducing high negative charge densities, enabling extreme swelling, robust pH/ion stimuli-responsiveness, and mimicking biomimetic heparin-like properties for wound healing. Finally, polymer grafting [72,88,107] represents the most sophisticated route, enabling multi-stimuli responsiveness and synergistic material properties, although it carries a significantly higher synthetic complexity and monomer purification burden. Choosing the optimal modification route thus requires a careful balance between the target clinical application, the necessary stimuli-responsiveness, and the associated regulatory purification demands.

## 6. Challenges and Future Perspectives

Despite the immense clinical and environmental promise of cellulose-based hydrogels, several critical challenges must be resolved to facilitate their widespread commercial translation, particularly within the domain of drug delivery systems.

### 6.1. Mechanical Weakness and the Softness-Strength Paradox

The first fundamental limitation is the inherent mechanical weakness of pristine polysaccharide hydrogels. Native cellulosic networks often exhibit pronounced brittleness and poor load-bearing capacity; as highlighted in patent literature, many unmodified biopolymer gels are so fragile they can be fractured by light mechanical touch [124]. For demanding applications such as artificial cartilage or muscle simulants, this mechanical deficit is a critical barrier. While increasing the chemical cross-link density or physically densifying the polymer network effectively enhances the elastic modulus, it does so at the severe expense of the equilibrium swelling ratio and matrix porosity. Consequently, researchers face a persistent design trade-off: balancing structural resilience against maintaining the high hydration and mesh size necessary for cellular infiltration and unhindered drug diffusion [125].

### 6.2. DDS-Specific Physicochemical and Translational Challenges

When applied specifically as drug carriers, cellulose-based hydrogels face distinct formulation and physical chemistry limitations:

- The Solubility and Burst-Release Challenge: Because cellulose derivatives are fundamentally hydrophilic, they exhibit poor loading efficiency for lipophilic or hydrophobic active pharmaceutical ingredients, which constitute approximately 70–80% of drug discovery pipelines [126]. Encapsulating these hydrophobic molecules often necessitates complex, multi-component carrier systems (such as lipid nanoparticles or cyclodextrin complexes) [127]. Furthermore, preventing the premature “burst release” of physically dispersed, weakly bound drugs remains a primary technical hurdle [128]. Achieving true, zero-order sustained release over extended periods requires precise thermodynamic tailoring of the drug-polymer affinity to prevent the drug from rapidly diffusing out during the initial swelling phase [129].
- The Sterilization Paradox: The clinical translation of any injectable or implantable DDS is strictly dependent on achieving sterility [130]. However, cellulose hydrogels and their loaded therapeutic agents are sensitive to conventional terminal sterilization methods. Thermal sterilization (autoclaving) can trigger polymer hydrolysis, matrix collapse, and the thermal inactivation of sensitive biologics [131]. Conversely, gamma-ray or electron-beam irradiation often induces polymer chain scission, radically altering the viscosity and release kinetics of the gel, while chemical sterilization (such as ethylene oxide) risks leaving toxic, non-biocompatible gaseous residues in the porous matrix [132]. Because of this complexity, sterilizing drug-loaded hydrogels requires an arduous, case-by-case optimization process, as there are no universal, standardized sterilization protocols [133].
- Regulatory Hurdles for Combination Products: From a regulatory standpoint, a cellulose hydrogel designed for controlled drug release is typically classified as a “drug-device combination product” or a “medical device with an ancillary drug substance”. Securing official regulatory approval (such as FDA clearance) is extremely complex. It requires exhaustive characterization of the hydrogel’s in vivo degradation rate, the systemic biodistribution of its degradation byproducts, and proof that the manufacturing process is reproducible under Good Manufacturing Practices [134].

### 6.3. Biocompatibility, Purification, and Environmental Desirability

Although native cellulose is biologically inert and highly biocompatible, the chemical modifications and covalent cross-linking agents used during hydrogel synthesis can introduce severe cytotoxicity if not rigorously managed. Residual unreacted monomers, organic solvents, or toxic cross-linkers must be thoroughly extracted prior to clinical use to ensure absolute cytocompatibility and safe in vivo biodegradability [135]. Overcoming these purification bottlenecks is essential to fulfilling the “circular economy” mandate. Currently, the global market for superabsorbents and medical dressings is dominated by non-degradable, petroleum-derived polyacrylic acids. Successfully scaling fully bio-based, rigorously purified, and degradable cellulose gels is therefore desirable as an environmental and ecological imperative [136].

### 6.4. Future Outlook: Smart Gels and Process Innovations

Looking ahead, the next frontier in this field is the development of fully integrated, “smart” cellulose nanocomposites. Researchers are increasingly embedding inorganic nanoparticles—such as gold nanoshells for photothermal therapy or iron oxide nanoparticles for magnetic hyperthermia—directly into the cellulosic backbone to enable externally triggered, on-demand drug release and localized sensing [137]. Concurrently, the incorporation of bioactive agents, such as antimicrobial peptides, growth factors, or drug-loaded liposomes, is transforming passive cellulose dressings into active, responsive therapeutic depots [138]. By grafting stimuli-responsive synthetic polymers (such as thermo-responsive poly(NIPAAm) or pH-sensitive polyacids) onto the cellulose scaffold, or by creating interpenetrating networks with other synergistic biopolymers like hyaluronic acid and chitosan, bioengineers are yielding advanced matrices that dynamically swell or shrink in response to specific physiological cues [139].

Despite their significant therapeutic promise, the transition of these stimuli-responsive “smart” systems from laboratory benches to commercial manufacturing is severely bottlenecked by thermodynamic, chemical, and operational constraints.

From a structural perspective, long-term stability remains a critical issue; cyclic swelling and deswelling transitions in response to fluctuating pH or temperature subject the macromolecular network to severe physical stress [139]. This repetitive expansion and contraction often triggers polymer fatigue, network hysteresis, and irreversible structural collapse of the cross-linked nodes over extended service lives.

Furthermore, achieving reliable batch-to-batch reproducibility is exceptionally difficult due to the inherent structural heterogeneity of native and semi-synthetic cellulose. Natural variations in polydispersity, molecular weight distribution, and the exact degree of substitution (DS) along the polysaccharide backbone mean that precise transition thresholds—such as the lower critical solution temperature of grafted poly(NIPAAm) chains—can fluctuate significantly between synthesis batches.

The industrial scalability of these responsive systems is limited by the chemical complexity of their synthesis. Fabricating responsive smart gels often relies on multi-step grafting reactions or host-guest supramolecular assemblies (e.g., azobenzene-cyclodextrin complexes) [88,107]. These pathways require strictly controlled reaction environments, organic solvents, and extensive, cost-prohibitive purification protocols to completely eliminate cytotoxic, unreacted synthetic monomers before clinical deployment. Addressing these physical-chemical bottlenecks is therefore prerequisite to establishing smart cellulose hydrogels as standardized, reproducible, and translationally viable clinical products.

Finally, breakthrough process innovations are controlled to develope the clinical applicability of cellulose gels. The transition toward greener, easily recoverable solvent systems is making large-scale production more viable. More importantly, the advent of 3D bioprinting and the electrospinning of nanofibrillated cellulose networks allow for the fabrication of patient-specific, anatomically precise scaffolds [140]. Literature is already forecasting the deployment of specialized cellulosic bioinks and 3D-printed “tunneling wound fillers” custom-designed for deep trauma [141]. As these advanced manufacturing technologies mature, functionally tailored cellulose hydrogels are positioned to become a gold standard in translational medicine, successfully achieving the twin goals of superior patient outcomes and global manufacturing sustainability.

### 6.5. Green Chemistry Assessment

The sustainability of cellulose hydrogels depends mainly on three technical factors: solvent efficiency, degradation pathways, and the carbon footprint relative to synthetic polymers. Regarding solvent recovery and energy consumption, the field is transitioning from complex ionic liquids—which are energy-intensive and difficult to recover—to aqueous NaOH/urea or solvent-free systems (e.g., cryogelation and radiation-induced cross-linking), which offer a significantly lower carbon footprint [142]. Ecological toxicity is inherently minimized by the biodegradability of the cellulose backbone, which undergoes enzymatic and hydrolytic degradation into non-toxic glucose units, as opposed to the persistent microplastic accumulation of petroleum-derived polyacrylates [143]. Standardized Life Cycle Assessment (LCA) data for these hydrogels is currently missing from the literature [144]. Comparisons of the ‘green potential’ between cellulose and synthetic benchmarks remain qualitative until the research community establishes uniform metrics for energy expenditure and ecological toxicity during industrial-scale synthesis.

### 6.6. Commercialization and Clinically Approved Cellulose-Based Formulations

To bridge the gap between laboratory innovation and clinical translation, it is essential to evaluate the current landscape of commercially available and clinically approved cellulose-based hydrogel formulations, namely hydroxypropyl cellulose, hydroxypropyl methylcellulose, carboxymethyl cellulose and sodium carboxymethylcellulose (NaCMC). Despite the stringent regulatory hurdles associated with combination devices and biopolymer scaling, several cellulose derivatives have successfully secured regulatory clearance and achieved widespread adoption in global medical markets. These established commercial platforms confirm that precise molecular and chemical engineering can successfully transition cellulose from an abundant raw material into highly trusted, clinically viable therapeutic devices.

In the domain of ophthalmic therapeutics, the clinical translation of cellulose is exemplified by Lacrisert^®^ (Bausch & Lomb), an FDA-approved sterile ophthalmic insert formulated from pure, preservative-free hydroxypropyl cellulose (HPC) [145]. This rod-shaped insert is placed directly into the inferior cul-de-sac of the eye, where it slowly hydrates, swells, and dissolves over several hours, acting as a dynamic, long-lasting tear film stabilizer to relieve severe dry eye syndrome.

The ophthalmic utility is further expanded by CuraGel^®^, (Curamed Opthalmics, Nieuwegein, The Netherlands) which leverages the exceptional rheological properties of medical-grade HPMC. Delivered in 2.0 mL pre-filled syringes, CuraGel operates as a high-viscosity ophthalmic viscoelastic device containing a specialized formula of 2.0% or 2.4% Plus, engineered to optimize zero shear rate viscosity while permitting unhindered manipulation during anterior segment surgeries. This high viscosity and elastic behavior are critical for maintaining anatomical spaces during complex ocular procedures. Beyond ophthalmology, the CuraGel platform has been adapted into transparent hydrogel wound dressings packaged in blister or double-pouch configurations, where the clear gel matrix provides a protective, moist environment that permits direct visual inspection of the healing bed without dressing removal [146].

For metabolic and gastrointestinal interventions, Plenity^®^ (Gelesis. Calimera, Italy) represents a highly successful commercial achievement, having secured FDA clearance as an orally administered weight management hydrogel [147]. Composed of CMC covalently cross-linked with naturally derived citric acid, these superabsorbent hydrogel particles are ingested in capsule form. Upon reaching the stomach, they rapidly expand to hundreds of times their dry volume to induce satiety before undergoing safe enzymatic degradation and excretion in the lower intestinal tract.

The clinical footprint of NaCMC is particularly dominant in advanced wound management, where maintaining a moist microenvironment is critical for autolytic debridement. Sterile, amorphous hydrogels such as Purilon^®^ Gel (Coloplast Corp, Minneapolis, MN, USA) [148] and Intrasite^®^ Gel (Smith & Nephew Healthcare Limited, Watford Hertfordshire, UK) leverage the water-binding capacity of NaCMC to gently rehydrate necrotic tissue, facilitate natural healing, and absorb excess slough [149]. Similarly, Aquacel^®^ (ConvaTec Global Corporate, London, UK) utilizes specialized NaCMC hydrofibers that instantly transition into a cohesive hydrogel sheet upon absorbing wound exudates, locking in bacteria and preventing tissue maceration [150].

This advanced wound management paradigm is complemented by the specialized hydrocolloid formulations developed under the DuoDerm^®^ (ConvaTec Global Corporate, London, UK) product line [147]. DuoDerm dressings are moisture-retentive barriers designed for partial and full-thickness wounds, including leg ulcers, pressure ulcers, traumatic injuries, donor sites, and partial-thickness burns. These devices utilize a multi-layered design where the outer waterproof layer consists of a polyurethane film or foam that insulates the wound from external bacterial or physical contaminants, while permitting normal washing and bathing. Beneath this protective shield, the inner hydrocolloidal layer comprises a synergistic blend of gelatin and carboxymethyl cellulose (CMC) that absorbs watery wound exudates to form a supportive, healing gel. To accommodate diverse anatomical and physical requirements, variations such as DuoDerm Extra Thin and DuoDerm Signal offer highly conformable, ultra-thin profiles ideal for visible or highly active, friction-prone areas like the face and hands. These hydrocolloid matrices can remain in place for up to a week, providing a painless, non-adherent removal process that prevents secondary trauma to the newly formed granulation tissue [151].

Collectively, these commercial benchmarks demonstrate that cellulose hydrogels are not merely theoretical concepts, but scalable, stable, and chemically predictable materials capable of meeting the rigorous requirements of modern clinical practice.

## 7. Conclusions

The growing intersection of advanced materials science, clinical pharmacology, and environmental sustainability has fundamentally elevated the role of biopolymers in modern medicine. As explored in this review, cellulose—the most abundant renewable polymer on Earth—provides an exceptionally versatile, hydrophilic platform for the engineering of high-performance hydrogels. By moving beyond the inherent insolubility and high crystallinity of native cellulose, researchers have established sophisticated physical, chemical, and hybrid cross-linking strategies. When coupled with targeted chemical modifications such as carboxylation, sulfation, and polymer grafting, these strategies allow for the precise tuning of the network’s mechanical elasticity, swelling kinetics, and localized stimuli-responsiveness.

Through comprehensive morphological, thermal, and rheological characterization, the structure–property relationships of these matrices have been clearly defined, paving the way for their successful deployment in critical biomedical applications. In the realm of drug delivery, the highly porous, hydrated 3D architecture of cellulose hydrogels prevents premature burst release and enables the sustained, site-specific delivery of both small-molecule therapeutics and delicate biologics. Simultaneously, their good biocompatibility, optical transparency, and exudate-absorbing capacities have solidified their value in advanced wound management and tissue engineering.

While formidable challenges remain—particularly the trade-off between mechanical robustness and hydration, the rigorous removal of toxic synthetic residues, and the economic hurdles of industrial scale-up—the trajectory of current research offers clear solutions. The integration of functional nanoparticles, the development of smart, stimuli-responsive interpenetrating networks, and the adoption of high-precision manufacturing techniques like 3D bioprinting represent the future of this field. Ultimately, the continuous refinement of sustainable, cellulose-based hydrogels stands as a testament to the fact that advanced clinical efficacy and rigorous environmental stewardship are not mutually exclusive, but rather synergistic goals in the future of biomedical engineering.

## Figures and Tables

**Table 1:** Comparative analysis of physical, chemical, and hybrid/mixed cross-linking strategies for cellulose-based hydrogels in biomedical applications.

Parameter	Physical Cross-Linking	Chemical Cross-Linking	Hybrid/IPN Cross-Linking
**Bonding Mechanism**	Reversible, non-covalent interactions (hydrogen bonds, ionic bridges, hydrophobic association)	Permanent, irreversible covalent bonding (ester, ether, or amide linkages)	Dual networks combining permanent covalent bonds and reversible physical entanglements
**Bond Stability**	Low to moderate; thermodynamically reversible and sensitive to physiological conditions	High; chemically stable and resistant to spontaneous physiological dissolution	Exceptionally high; synergistic stabilization of both networks
**Mechanical Strength**	Low to moderate; prone to plastic deformation and brittle under continuous load	High; superior elastic modulus (*G′*) and structural stability under load	Outstanding; high toughness, excellent elasticity, and dynamic cyclic compression stability
**Swelling Capacity**	High; network expands easily but is susceptible to premature dissolution	Moderate to high; inversely proportional to cross-link density	Highly tunable; retains substantial water within the double-network scaffold
**Toxicity Risk**	Minimal; typically avoids toxic organic solvents or chemical cross-linkers	Moderate to high; requires rigorous post-synthesis purification to remove unreacted cross-linkers	Low to moderate; depends on the nature of the secondary synthetic monomer and purification steps
**Typical Cellulose Examples**	Freeze–thaw CNC/gelatin gels [81]; Topical physical hydrogels [98]	EGDE-mediated covalent cross-linking of cationic HEC [85]; Gamma-radiation cross-linked CMC/EGDE acrylic acid [83]; Covalent CMC composite systems [68]	PAAm/CMC cryogels [72]; Double-layer hydrogels (Alginate/CMC core) [86]; Succinoglycan/CMC IPNs [87]

**Table 2:** Comparison of advantages, disadvantages, and structural impacts of cellulose chemical modification strategies in drug delivery.

Modification Strategy	Etherification	Carboxylation/Sulfation	Polymer Grafting
**Introduced Groups**	Alkyl, hydroxyalkyl, or carboxyalkyl groups (e.g., −CH_3_, —−CH_2_CH_2_OH)	Carboxyl (-COO^−^) or sulfate ester (−OSO_3_^2−^) groups	Synthetic polymer chains (e.g., poly(NIPAAm), polyacrylamide, poly(HEMA))
**Key Advantages**	Disrupts dense intra-chain hydrogen bonding; Dramatically enhances water solubility; Introduces thermal gelation profiles (HPMC, MC)	Introduces high negative charge density for extreme swelling; Imparts pH- and ion-responsiveness; Sulfation provides biomimetic heparin-like bioactivity	Integrates highly complex, multi-stimuli responsiveness (e.g., temperature and pH); Synergizes synthetic mechanical toughness with natural biocompatibility
**Main Disadvantages**	Requires harsh alkaline conditions and alkylating agents; Does not inherently introduce stimuli-responsive ionic charges	High electrostatic repulsion can cause excessive swelling and matrix dissolution if cross-linking is insufficient	Requires free-radical initiator systems; Presents high risk of residual, toxic unreacted monomer contamination
**Structural & Crystalline Impact**	Disrupts native Cellulose I crystallinity, rendering the polymer amorphous and highly soluble	Variable; sulfation can selectively functionalize the surface of nanocellulose (CNF/CNC) while preserving the crystalline core	Reorganizes the crystalline domains into a highly amorphous, functional hybrid network
**Typical Biomedical Examples**	In-situ gelling ophthalmic delivery systems (Carbopol/MC) [93,94]; Metronidazole-loaded solid lipid nanoparticles in HEC gels [101]	Gastric-targeted oral DDS (CMC/LDH beads) [82]; Lyophilized NaCMC antimicrobial wafers [102]; Topical bioadhesive dermal dressings [71]	Tough, covalently cross-linked PAAm/CMC cryogels [72]; Thiolated CMC microgels for synergistic cancer therapy [107]; Photo-switchable/self-healing Azobenzene-grafted CMC [88]

## Data Availability

No new data were produced.

## References

[1] Tofanica B.-M., Belosinschi D., Volf I. (2022). Gels, aerogels and hydrogels: A challenge for the Cellulose-Based product industries. Gels.

[2] Malekmohammadi S., Sedghi Aminabad N., Sabzi A., Zarebkohan A., Razavi M., Vosough M., Bodaghi M., Maleki H. (2021). Smart and Biomimetic 3D and 4D Printed Composite Hydrogels: Opportunities for Different Biomedical Applications. Biomedicines.

[3] Segneanu A.-E., Bejenaru L.E., Bejenaru C., Blendea A., Mogoşanu G.D., Biţă A., Boia E.R. (2025). Advancements in Hydrogels: A Comprehensive Review of Natural and Synthetic Innovations for Biomedical Applications. Polymers.

[4] Kausar A. (2020). Poly(acrylic acid) nanocomposites: Design of advanced materials. J. Plast. Film Sheeting.

[5] Hayes G., Laurel M., MacKinnon D., Zhao T., Houck H.A., Becer C.R. (2022). Polymers without Petrochemicals: Sustainable Routes to Conventional Monomers. Chem. Rev..

[6] Zare M., Zare M., Bigham A., Zare M., Zare M., Luo H., Ghomi E.R., Ramakrishna S. (2021). PHEMA: An Overview for Biomedical Applications. Int. J. Mol. Sci..

[7] Malafeev K. (2025). The Cytotoxicity of Biodegradable Microplastics and Nanoplastics: Current Status and Research Prospects. Microplastics.

[8] Leon-Bejarano M., Santos-Sauceda I., Dórame-Miranda R.F., Medina-Juárez L.Á., Gámez-Meza N., García-Galaz A., Simsek S., Ovando-Martínez M. (2023). Characterization of OSA starch-based films with nut-byproducts extracts for potential application as natural wound dressing. Polym. Bull..

[9] Ungureanu E., Mikhailidi A., Tofanica B.-M., Fortună M.E., Rotaru R., Ungureanu O.C., Samuil C., Popa V.I. (2025). Sustainable Gels from Polysaccharides in Agriculture. Polysaccharides.

[10] Tofanica B.-M., Callone E., Ungureanu E., Ungureanu O.C., Popa V.I. (2025). Structure of Cellulose Isolated from Rapeseed Stalks. Polymers.

[11] Dupont A.-L. (2003). Cellulose in lithium chloride/N,N-dimethylacetamide, optimisation of a dissolution method using paper substrates and stability of the solutions. Polymer.

[12] Acharya S., Liyanage S., Parajuli P., Rumi S.S., Shamshina J.L., Abidi N. (2021). Utilization of cellulose to its full potential: A review on cellulose dissolution, regeneration, and applications. Polymers.

[13] Mikhailidi A., Volf I., Belosinschi D., Tofanica B.-M., Ungureanu E. (2023). Cellulose-Based Metallogels—Part 1: Raw Materials and Preparation. Gels.

[14] Bhuyan M.M., Jeong J.-H. (2025). Preparation of Hydrogel by Crosslinking and Multi-Dimensional Applications. Gels.

[15] Cao L., Zhu J., Deng B., Zeng F., Wang S., Ma Y., Qin C., Yao S. (2022). Efficient swelling and mercerization of bagasse fiber by Freeze-Thaw-Assisted alkali treatment. Front. Energy Res..

[16] Wang M., Jiang G., Guo X., Zeng S., Zhao D. (2025). Cellulose Functional Gels: Physical design and promising applications. Adv. Phys. Res..

[17] Kahya N. (2025). Biocompatible CMC-based hydrogel fiber systems: Swelling control via trivalent ionic crosslinking. J. Polym. Res..

[18] Chen Y.M., Sun L., Yang S.A., Shi L., Zheng W.J., Wei Z., Hu C. (2017). Self-healing and photoluminescent carboxymethyl cellulose-based hydrogels. Eur. Polym. J..

[19] Li P., Liu R. (2015). Cellulose gels and microgels: Synthesis, service, and supramolecular interactions. Supramolecular Polymer Networks and Gels; Advances in Polymer Science.

[20] De Lima G.F., De Souza A.G., Rosa D.D.S. (2020). Nanocellulose as reinforcement in carboxymethylcellulose superabsorbent nanocomposite hydrogels. Macromol. Symp..

[21] Dudeja I., Mankoo R.K., Singh A., Kaur J. (2023). Citric acid: An ecofriendly cross-linker for the production of functional biopolymeric materials. Sustain. Chem. Pharm..

[22] Chang C., Duan B., Cai J., Zhang L. (2009). Superabsorbent hydrogels based on cellulose for smart swelling and controllable delivery. Eur. Polym. J..

[23] Ahmed M.S., Islam M., Hasan M.K., Nam K.-W. (2024). A Comprehensive Review of Radiation-Induced Hydrogels: Synthesis, Properties, and Multidimensional Applications. Gels.

[24] Kansai Research Institute KRI Inc (2025). Sulfated Esterified Cellulose Nanofiber and Dry Matter Thereof. Japan Patent.

[25] Tang Y., Fang Z., Lee H.-J. (2024). Exploring Applications and Preparation Techniques for Cellulose Hydrogels: A Comprehensive Review. Gels.

[26] Ciolacu D., Rudaz C., Vasilescu M., Budtova T. (2016). Physically and chemically cross-linked cellulose cryogels: Structure, properties and application for controlled release. Carbohydr. Polym..

[27] Shahi F., Zarei S., Othman R.S., Afshar H., Kamran F., Taromi A.A., Khonakdar H.A. (2025). Interpenetrating polymer networks in biomedical Fields: Recent advanced and applications. Polym. Adv. Technol..

[28] Rumon M.H. (2025). Advances in cellulose-based hydrogels: Tunable swelling dynamics and their versatile real-time applications. RSC Adv..

[29] Li Z., Wang M., Jiang G., Zhou J., Li D., Zhang W., Zhao D. (2026). Smart molecular design for functional cellulose gels and flexible devices. Smart Mol..

[30] Cuissinat C., Navard P., Heinze T. (2007). Swelling and dissolution of cellulose, Part V: Cellulose derivatives fibres in aqueous systems and ionic liquids. Cellulose.

[31] Xue X., Hu J., Guo P., Wang L., Wang L., Dong Y., Xiao F., Li C., Ding S. (2026). Cellulose-Based Composite Hydrogels for Heavy Metal Ion Removal: Recent Advances and Engineering Perspectives. Gels.

[32] He M., Lin Y., Huang Y., Fang Y., Xiong X. (2025). Research Progress of the Preparation of Cellulose Ethers and Their Applications: A Short Review. Molecules.

[33] Li B., Xu C., Yu J., Liu L., Zhang X., Fan Y. (2023). One-pot cellulose etherification and self-crosslinking via a mild hydroxyl–yne click reaction in a homogeneous system. Green Chem..

[34] Kayra N., Aytekin A.Ö. (2018). Synthesis of Cellulose-Based Hydrogels: Preparation, formation, Mixture, and Modification. Cellulose-Based Superabsorbent Hydrogels; Polymers and Polymeric Composites.

[35] Büyüküstün A.D., Erişir E., Gümüşkaya E. (2025). Swelling Capacity in Carboxymethylcellulose-Cellulose Hybrid Hydrogels: The Effects of Oxidation with Zinc Chloride and Refining on Cellulose Used as Reinforcement. Drew. Pr. Nauk. Doniesienia Komun. = Wood Res. Pap. Rep. Announc..

[36] Alavarse A.C., Frachini E.C.G., Da Silva R.L.C.G., Lima V.H., Shavandi A., Petri D.F.S. (2022). Crosslinkers for polysaccharides and proteins: Synthesis conditions, mechanisms, and crosslinking efficiency, a review. Int. J. Biol. Macromol..

[37] Seddiqi H., Oliaei E., Honarkar H., Jin J., Geonzon L.C., Bacabac R.G., Klein-Nulend J. (2021). Cellulose and its derivatives: Towards biomedical applications. Cellulose.

[38] Kazachenko A.S., Vasilieva N.Y., Berezhnaya Y.D., Fetisova O.Y., Borovkova V.S., Malyar Y.N., Sudakova I.G., Sychev V.V., Issaoui N., Lutoshkin M.A. (2023). Sulfation of Birch Wood Microcrystalline Cellulose with Sulfamic Acid Using Ion-Exchange Resins as Catalysts. Polymers.

[39] Jaouahar M., Ablouh E.-H., Hanani Z., Jaklič B., Spreitzer M., Semlali F.-Z., Benhamou A.A., Samih Y., Achaby M.E., Sehaqui H. (2024). Preparation and characterization of sulfated nanocellulose: From hydrogels to highly transparent films. Int. J. Biol. Macromol..

[40] Sharma K., Choudhary P., Majeed A., Guleria S., Kumar M., Rana A.K., Rajauria G. (2025). Cellulose based membranes, hydrogels and aerogels for water treatment application. Ind. Crops Prod..

[41] Peschel D., Zhang K., Aggarwal N., Brendler E., Fischer S., Groth T. (2009). Synthesis of novel celluloses derivatives and investigation of their mitogenic activity in the presence and absence of FGF2. Acta Biomater..

[42] Zhang K., Peschel D., Bäucker E., Groth T., Fischer S. (2010). Synthesis and characterisation of cellulose sulfates regarding the degrees of substitution, degrees of polymerisation and morphology. Carbohydr. Polym..

[43] Bashir S., Hina M., Iqbal J., Rajpar A.H., Mujtaba M.A., Alghamdi N.A., Wageh S., Ramesh K., Ramesh S. (2020). Fundamental Concepts of Hydrogels: Synthesis, Properties, and Their Applications. Polymers.

[44] Zhang X., Wang Y., Zhao J., Xiao M., Zhang W., Lu C. (2016). Mechanically strong and thermally responsive cellulose Nanofibers/Poly(N-isopropylacrylamide) composite aerogels. ACS Sustain. Chem. Eng..

[45] Sirviö J.A., Ukkola J., Liimatainen H. (2019). Direct sulfation of cellulose fibers using a reactive deep eutectic solvent to produce highly charged cellulose nanofibers. Cellulose.

[46] Protsak I.S., Morozov Y.M. (2025). Fundamentals and Advances in Stimuli-Responsive Hydrogels and Their Applications: A Review. Gels.

[47] Alonso-Simón A., García-Angulo P., Mélida H., Encina A., Álvarez J.M., Acebes J.L. (2011). The use of FTIR spectroscopy to monitor modifications in plant cell wall architecture caused by cellulose biosynthesis inhibitors. Plant Signal. Behav..

[48] Salem K.S., Kasera N.K., Rahman M.A., Jameel H., Habibi Y., Eichhorn S.J., French A.D., Pal L., Lucia L.A. (2023). Comparison and assessment of methods for cellulose crystallinity determination. Chem. Soc. Rev..

[49] Pa’e N., Salehudin M.H., Hassan N.D., Marsin A.M., Muhamad I.I. (2018). Thermal Behavior of Bacterial Cellulose Based Hydrogels with Other Composites and Related Instrumental Analysis. Cellulose-Based Superabsorbent Hydrogels; Polymers and Polymeric Composites.

[50] Martinez-Garcia F.D., Fischer T., Hayn A., Mierke C.T., Burgess J.K., Harmsen M.C. (2022). A Beginner’s Guide to the Characterization of Hydrogel Microarchitecture for Cellular Applications. Gels.

[51] Stojkov G., Niyazov Z., Picchioni F., Bose R.K. (2021). Relationship between Structure and Rheology of Hydrogels for Various Applications. Gels.

[52] Chen M.H., Wang L.L., Chung J.J., Kim Y.-H., Atluri P., Burdick J.A. (2017). Methods to assess Shear-Thinning hydrogels for application as injectable biomaterials. ACS Biomater. Sci. Eng..

[53] Palma D., Lagos O., Souto C., Pérez A., Quezada L., Hirzel J., Vera M., Ulloa J., Urbano B. (2024). Evaluation of a Natural Superabsorbent Polymer on Water Retention Capacity in Coarse-Textured Soils. Water.

[54] Guvendiren M., Yang S., Burdick J.A. (2009). Swelling-Induced Surface Patterns in Hydrogels with Gradient Crosslinking Density. Adv. Funct. Mater..

[55] Ticknor L.B. (1963). A thermodynamic method for the comparison of the order distribution in cellulose and in other polymers. J. Polym. Sci. Part A Gen. Pap..

[56] Li Z., Zhang M. (2023). Progress in the Preparation of Stimulus-Responsive Cellulose Hydrogels and Their Application in Slow-Release Fertilizers. Polymers.

[57] Kamaly N., Yameen B., Wu J., Farokhzad O.C. (2016). Degradable Controlled-Release Polymers and Polymeric nanoparticles: Mechanisms of controlling drug release. Chem. Rev..

[58] Nagay B.E., Janghour L.M., El-Khordagui L.K., Akhavan B., Barão V.A.R., Dananjaya V., Abeykoon C., El-Habashy S.E., Dodda J.M. (2026). Multifunctional implantable hydrogels: Smart platforms at the forefront of biomedical innovation. Mater. Today Bio.

[59] Deng Y., Zhu T., Cheng Y., Zhao K., Meng Z., Huang J., Cai W., Lai Y. (2024). Recent Advances in Functional Cellulose-Based Materials: Classification, properties, and Applications. Adv. Fiber Mater..

[60] Ciolacu D.E., Nicu R., Ciolacu F. (2020). Cellulose-Based Hydrogels as Sustained Drug-Delivery Systems. Materials.

[61] Sun B., Zhang M., Shen J., He Z., Fatehi P., Ni Y. (2018). Applications of cellulose-based materials in sustained drug delivery systems. Curr. Med. Chem..

[62] Kavitha A.A., Paul K.T., Anilkumar P. (2020). Cellulose-derived materials for drug delivery applications. Elsevier eBooks.

[63] Luo D., Wang Y., Zhou D., Wang S., Guo M. (2026). Cellulose and its derivatives in drug delivery: Recent advances and applications. Pharmaceutics.

[64] Garg T., Arora S., Pahwa R. (2025). Cellulose and its derivatives: Structure, modification, and application in controlled drug delivery. Future J. Pharm. Sci..

[65] Kamel S., Ali N., Jahangir K., Shah S.M., El-Gendy A.A. (2008). Pharmaceutical significance of cellulose: A review. eXPRESS Polym. Lett..

[66] Khiste R., Bhapkar N., Kulkarni N. (2021). A review on applications of Hydroxy propyl methyl cellulose and natural polymers for the development of modified release drug delivery systems. Res. J. Pharm. Technol..

[67] Majumdar S. (2025). Cellulose-based hydrogels for drug delivery applications. Elsevier eBooks.

[68] Gupta Y., Khan M.S., Bansal M., Singh M.K., Pragatheesh K., Thakur A. (2024). A review of carboxymethyl cellulose composite-based hydrogels in drug delivery applications. Results Chem..

[69] Majumder T., Biswas G.R., Majee S.B. (2016). Hydroxy propyl methyl cellulose: Different aspects in drug delivery. J. Pharm. Pharmacol..

[70] Colombo P., Bettini R., Catellani P.L., Santi P., Peppas N.A. (1999). Drug volume fraction profile in the gel phase and drug release kinetics in hydroxypropylmethyl cellulose matrices containing a soluble drug. Eur. J. Pharm. Sci..

[71] Jones D.S., Woolfson A.D., Brown A.F. (1997). Textural, viscoelastic and mucoadhesive properties of pharmaceutical gels composed of cellulose polymers. Int. J. Pharm..

[72] Viboonratanasri D., King D.R., Okumura T., Terkawi M.A., Katsuyama Y., Lama M., Yasui T., Kurokawa T. (2025). Porous and tough Polyacrylamide/Carboxymethyl cellulose gels chemically crosslinked via Cryo-UV polymerization for sustained drug release. Gels.

[73] Jeong D., Joo S.-W., Hu Y., Shinde V.V., Cho E., Jung S. (2018). Carboxymethyl cellulose-based superabsorbent hydrogels containing carboxymehtyl β-cyclodextrin for enhanced mechanical strength and effective drug delivery. Eur. Polym. J..

[74] Treesuppharat W., Rojanapanthu P., Siangsanoh C., Manuspiya H., Ummartyotin S. (2017). Synthesis and characterization of bacterial cellulose and gelatin-based hydrogel composites for drug-delivery systems. Biotechnol. Rep..

[75] Liu Z., Zhang S., He B., Wang S., Kong F. (2021). Synthesis of cellulose aerogels as promising carriers for drug delivery: A review. Cellulose.

[76] García-González C.A., Alnaief M., Smirnova I. (2011). Polysaccharide-based aerogels—Promising biodegradable carriers for drug delivery systems. Carbohydr. Polym..

[77] Gelas L., Budtova T. (2024). From cellulose solutions to aerogels and xerogels: Controlling properties for drug delivery. Biomacromolecules.

[78] Dai L., Si C. (2018). Recent advances on Cellulose-Based Nano-Drug delivery systems: Design of prodrugs and nanoparticles. Curr. Med. Chem..

[79] Gupta B., Mishra V., Gharat S., Momin M., Omri A. (2021). Cellulosic polymers for enhancing drug bioavailability in ocular drug delivery systems. Pharmaceuticals.

[80] Hosny K.M., Alkhalidi H.M., Alharbi W.S., Md S., Sindi A.M., Ali S.A., Bakhaidar R.B., Almehmady A.M., Alfayez E., Kurakula M. (2021). Recent trends in assessment of cellulose derivatives in designing novel and Nanoparticulate-Based drug delivery systems for improvement of oral health. Polymers.

[81] Ooi S.Y., Ahmad I., Mohd Amin C.I.M. (2015). Cellulose nanocrystals extracted from rice husks as a reinforcing material in gelatin hydrogels for use in controlled drug delivery systems. Ind. Crops Prod..

[82] Barkhordari S., Yadollahi M., Namazi H. (2014). PH sensitive nanocomposite hydrogel beads based on carboxymethyl cellulose/layered double hydroxide as drug delivery systems. J. Polym. Res..

[83] Ali A.E., El-Rehim H.a.A., Kamal H., Hegazy D.E.A. (2008). Synthesis of carboxymethyl cellulose based drug carrier hydrogel using ionizing radiation for possible use as site specific delivery system. J. Macromol. Sci. Part A.

[84] Amin M.C.I.M., Ahmad N., Halib N., Ahmad I. (2011). Synthesis and characterization of thermo- and pH-responsive bacterial cellulose/acrylic acid hydrogels for drug delivery. Carbohydr. Polym..

[85] Rodríguez R., Alvarez-Lorenzo C., Concheiro A. (2003). Cationic cellulose hydrogels: Kinetics of the cross-linking process and characterization as pH-/ion-sensitive drug delivery systems. J. Control. Release.

[86] Hu Y., Hu S., Zhang S., Dong S., Hu J., Kang L., Yang X. (2021). A double-layer hydrogel based on alginate-carboxymethyl cellulose and synthetic polymer as sustained drug delivery system. Sci. Rep..

[87] Shin Y., Kim D., Hu Y., Kim Y., Hong I.K., Kim M.S., Jung S. (2021). pH-Responsive Succinoglycan-Carboxymethyl Cellulose Hydrogels with Highly Improved Mechanical Strength for Controlled Drug Delivery Systems. Polymers.

[88] Kim Y., Jeong D., Shinde V.V., Hu Y., Kim C., Jung S. (2020). Azobenzene-grafted carboxymethyl cellulose hydrogels with photo-switchable, reduction-responsive and self-healing properties for a controlled drug release system. Int. J. Biol. Macromol..

[89] Jeong D., Kim H.K., Jeong J.-P., Dindulkar S.D., Cho E., Yang Y.-H., Jung S. (2016). Cyclosophoraose/cellulose hydrogels as an efficient delivery system for galangin, a hydrophobic antibacterial drug. Cellulose.

[90] Lotfy V.F., Basta A.H. (2020). Optimizing the chitosan-cellulose based drug delivery system for controlling the ciprofloxacin release versus organic/inorganic crosslinker, characterization and kinetic study. Int. J. Biol. Macromol..

[91] Xu J., Tan X., Chen L., Li X., Xie F. (2019). Starch/microcrystalline cellulose hybrid gels as gastric-floating drug delivery systems. Carbohydr. Polym..

[92] Javanbakht S., Shaabani A. (2019). Carboxymethyl cellulose-based oral delivery systems. Int. J. Biol. Macromol..

[93] Sultana Y., Aqil M., Ali A., Zafar S. (2006). Evaluation of Carbopol-Methyl Cellulose based Sustained-Release Ocular delivery System for pefloxacin mesylate using Rabbit Eye model. Pharm. Dev. Technol..

[94] Kumar S., Haglund B.O., Himmelstein K.J. (1994). In situ -Forming gels for ophthalmic drug delivery. J. Ocul. Pharmacol. Ther..

[95] Kolawole O.M., Cook M.T. (2023). In situ gelling drug delivery systems for topical drug delivery. Eur. J. Pharm. Biopharm..

[96] Vlaia L., Coneac G., Olariu I., Vlaia V., Lupuleasa D. (2016). Cellulose-Derivatives-Based hydrogels as vehicles for dermal and transdermal drug delivery. InTech eBooks.

[97] Patil P.B., Datir S.K., Saudagar R.B. (2019). A review on topical gels as drug delivery system. J. Drug Deliv. Ther..

[98] Ribeiro A.M., Magalhães M., Veiga F., Figueiras A. (2018). Cellulose-Based hydrogels in topical drug delivery: A challenge in medical devices. Cellulose-Based Superabsorbent Hydrogels; Polymers and Polymeric Composites.

[99] Estabragh M.A.R., Bami M.S., Dehghannoudeh G., Noudeh Y.D., Moghimipour E. (2023). Cellulose derivatives and natural gums as gelling agents for preparation of emulgel-based dosage forms: A brief review. Int. J. Biol. Macromol..

[100] Bruno L., Kasapis S., Chaudhary V., Chow K.T., Heng P.W.S., Leong L.P. (2011). Temperature and time effects on the structural properties of a non-aqueous ethyl cellulose topical drug delivery system. Carbohydr. Polym..

[101] Ho H.N., Le H.H., Le T.G., Duong T.H.A., Ngo V.Q.T., Dang C.T., Nguyen V.M., Tran T.H., Nguyen C.N. (2021). Formulation and characterization of hydroxyethyl cellulose-based gel containing metronidazole-loaded solid lipid nanoparticles for buccal mucosal drug delivery. Int. J. Biol. Macromol..

[102] Ng S.-F., Jumaat N. (2013). Carboxymethyl cellulose wafers containing antimicrobials: A modern drug delivery system for wound infections. Eur. J. Pharm. Sci..

[103] Cui N., Xu Z., Zhao X., Yuan M., Pan L., Lu T., Du A., Qin L. (2022). In vivo effect of Resveratrol-Cellulose Aerogel drug delivery system to relieve inflammation on sports osteoarthritis. Gels.

[104] Han W., Liu F., Li Y., Liu G., Li H., Xu Y., Sun S. (2023). Advances in natural Polymer-Based transdermal drug delivery systems for tumor therapy. Small.

[105] Yusefi M., Soon M.L.-K., Teow S.-Y., Monchouguy E.I., Neerooa B.N.H.M., Izadiyan Z., Jahangirian H., Rafiee-Moghaddam R., Webster T.J., Shameli K. (2022). Fabrication of cellulose nanocrystals as potential anticancer drug delivery systems for colorectal cancer treatment. Int. J. Biol. Macromol..

[106] Yusefi M., Shameli K., Lee-Kiun M.S., Teow S.-Y., Moeini H., Ali R.R., Kia P., Jie C.J., Abdullah N.H. (2023). Chitosan coated magnetic cellulose nanowhisker as a drug delivery system for potential colorectal cancer treatment. Int. J. Biol. Macromol..

[107] Zhou J., Zhang Z., Zhang Z., Wu T., Li H., Hao X., Liu X., Gong T., Liu D., Wei S. (2025). A facile dual-drug delivery system using cellulose-based microgel/hydrogel for enhanced gastric cancer therapy. Colloids Surf. B Biointerfaces.

[108] Abeer M.M., Amin M.C.I.M., Martin C. (2014). A review of bacterial cellulose-based drug delivery systems: Their biochemistry, current approaches and future prospects. J. Pharm. Pharmacol..

[109] Numata Y., Mazzarino L., Borsali R. (2015). A slow-release system of bacterial cellulose gel and nanoparticles for hydrophobic active ingredients. Int. J. Pharm..

[110] Chung C.K., Beekmann U., Kralisch D., Bierau K., Chan A., Ossendorp F., Cruz L.J. (2022). Bacterial cellulose as drug delivery system for optimizing release of immune checkpoint blocking antibodies. Pharmaceutics.

[111] Bora N.S., Dutta K.N., Talukder A., Gogoi B., Gam S., Deka K., Deka G., Sahariah B.J. (2025). Cellulose-based hydrogels for wound dressing and wound healing applications. Cellulose-Based Hydrogels.

[112] Negut I., Visan A.I. (2026). Cellulose-Based Hydrogels for Chronic Wound Healing: Bridging Biomaterial Design and Clinical Unmet Needs. Gels.

[113] Bukatuka C.F., Mbituyimana B., Xiao L., Qaed Ahmed A.A., Qi F., Adhikari M., Shi Z., Yang G. (2025). Recent Trends in the Application of Cellulose-Based Hemostatic and Wound Healing Dressings. J. Funct. Biomater..

[114] Ribeiro M., Simões M., Vitorino C., Mascarenhas-Melo F. (2024). Hydrogels in Cutaneous Wound Healing: Insights into Characterization, Properties, Formulation and Therapeutic Potential. Gels.

[115] Long Y., Dimde M., Adler J.M., Vidal R.M., Povolotsky T.L., Nickl P., Achazi K., Trimpert J., Kaufer B.B., Haag R. (2024). Sulfated Cellulose Nanofiber Hydrogel with Mucus-Like Activities for Virus Inhibition. ACS Appl. Mater. Interfaces.

[116] Vázquez-Rivas E., Desales-Guzmán L.A., Pacheco-Sánchez J.H., Burillo-Amezcua S.G. (2025). Cellulose-Based Hybrid Hydrogels for Tissue Engineering Applications: A Sustainable Approach. Gels.

[117] Tong Y., Cai Y., Wu Y., Zhuo W., Liao J. (2026). From Design to Application: Advanced Cellulose Scaffolds for Engineered Tissue Regeneration. Polymers.

[118] Lazarus E., Bermudez-Lekerika P., Farchione D., Schofield T., Howard S., Mambetkadyrov I., Lamoca M., Rivero I.V., Gantenbein B., Lewis C.L. (2021). Sulfated Hydrogels in Intervertebral Disc and Cartilage Research. Cells.

[119] Cordeiro R., Alvites R.D., Sousa A.C., Lopes B., Sousa P., Maurício A.C., Alves N., Moura C. (2023). Cellulose-Based Scaffolds: A Comparative Study for Potential Application in Articular Cartilage. Polymers.

[120] Wu K.Y., Akbar D., Giunta M., Kalevar A., Tran S.D. (2024). Hydrogels in Ophthalmology: Novel Strategies for Overcoming Therapeutic Challenges. Materials.

[121] Nguyen L.R. (2011). Review of hydroxypropyl cellulose ophthalmic inserts for treatment of dry eye. Clin. Ophthalmol..

[122] Quazi M.Z., Hwang J., Song Y., Park N. (2023). Hydrogel-Based Biosensors for Effective Therapeutics. Gels.

[123] Shen C., Wang Y., Xiao X., Yang R., Chen H., Yuan P., Zhang Y., Lyu G., Shin J., Chen G. (2026). Hydrogels for in vivo biomedical applications: Recent advances and future perspectives. J. Mater. Chem. B.

[124] Dutt K.R., Roy A., Haque A., Hasan N., Deep M.P., Kumar M., Singh S., Kumari S., Sharma S., Kumar A. (2024). Overcoming the limitations of hydrogels: Exploring superior materials for improved mechanical and functional efficiency. Int. J. Pharm. Sci..

[125] Baumgartner S., Kristl J., Peppas N.A. (2002). Network structure of cellulose ethers used in pharmaceutical applications during swelling and at equilibrium. Pharm. Res..

[126] Rosenzweig O., Lavy E., Gati I., Kohen R., Friedman M. (2013). Development andin vitrocharacterization of floating sustained-release drug delivery systems of polyphenols. Drug Deliv..

[127] Huang R., Song H., Li S., Guan X. (2024). Selection strategy for encapsulation of hydrophilic and hydrophobic ingredients with food-grade materials: A systematic review and analysis. Food Chem. X.

[128] Yang M., Zhang Y., Liu Z., Liu L., Wang X., Qian L. (2023). Advances in research on cellulose-based drug carriers. Pap. Biomater..

[129] Li X., Li Q., Zhao C. (2021). Zero-Order controlled release of Water-Soluble drugs using a marker pen platform. ACS Omega.

[130] Tao M., Ao T., Mao X., Yan X., Javed R., Hou W., Wang Y., Sun C., Lin S., Yu T. (2021). Sterilization and disinfection methods for decellularized matrix materials: Review, consideration and proposal. Bioact. Mater..

[131] Bakhrushina E.O., Afonina A.M., Mikhel I.B., Demina N.B., Plakhotnaya O.N., Belyatskaya A.V., Krasnyuk I.I., Krasnyuk I.I. (2024). Role of Sterilization on In Situ Gel-Forming Polymer Stability. Polymers.

[132] Moore E., Cortese Y.J., Colbert D.M. (2025). A Review of Sterilization Methods and Their Commercial Impacts on Polysaccharide-Based Biomaterials. Macromol.

[133] Sintzel M.B., Merkli A., Tabatabay C., Gurny R. (1997). Influence of Irradiation sterilization on Polymers Used as Drug Carriers—A Review. Drug Dev. Ind. Pharm..

[134] Lu P., Ruan D., Huang M., Tian M., Zhu K., Gan Z., Xiao Z. (2024). Harnessing the potential of hydrogels for advanced therapeutic applications: Current achievements and future directions. Signal Transduct. Target. Ther..

[135] Yao P., Deng X., Zhang R., Liu Z. (2026). Hydrogel-Based Implantable Medical Devices: Pioneering therapies and monitoring. Adv. Funct. Mater..

[136] Venturelli G., Villa F., Petraretti M., Guagliano G., Levi M., Petrini P. (2025). Bacterial Cellulose for Scalable and Sustainable Bio-Gels in the Circular Economy. Gels.

[137] Chuang Y.-C., Lee H.-L., Chiou J.-F., Lo L.-W. (2022). Recent Advances in Gold Nanomaterials for Photothermal Therapy. J. Nanotheranostics.

[138] Pang Q., Jiang Z., Wu K., Hou R., Zhu Y. (2023). Nanomaterials-Based Wound Dressing for Advanced Management of Infected Wound. Antibiotics.

[139] Babelyte M., Peciulyte L., Navikaite-Snipaitiene V., Bendoraitiene J., Samaryk V., Rutkaite R. (2023). Synthesis and Characterization of Thermoresponsive Chitosan-graft-poly(N-isopropylacrylamide) Copolymers. Polymers.

[140] Yücer S., Sarac B., Ciftci F. (2025). Bioprinting revolution: Innovative design of 3D bioactive scaffolds for living organs and transdermal tissues. Bioeng. Transl. Med..

[141] Govindharaj M., Hashimi N.A., Soman S.S., Zhou J., AlAwadhi S., Vijayavenkataraman S. (2024). 3D-bioprinted tri-layered cellulose/collagen-based drug-eluting fillers for the treatment of deep tunneling wounds. Bio-Des. Manuf..

[142] Cai J., Zhang L., Zhou J., Li H., Chen H., Jin H. (2004). Novel Fibers Prepared from Cellulose in NaOH/Urea Aqueous Solution. Macromol. Rapid Commun..

[143] Ehrhardt A., Bui H.M., Duelli H., Bechtold T. (2009). NaOH/urea aqueous solutions improving properties of regenerated-cellulosic fabrics. J. Appl. Polym. Sci..

[144] Conesa J.A., Córdoba V.N. (2024). Recovery of Cellulose Contained in Mixed Fabrics. Processes.

[145] Luchs J.I., Nelinson D.S., Macy J.I. (2010). Efficacy of hydroxypropyl cellulose ophthalmic inserts (LACRISERT) in subsets of patients with dry eye syndrome: Findings from a patient registry. Cornea.

[146] Yadav R., Tiwari N., Nainwal L.M., Hasan N., Kumar N., Kumar V. (2025). Exploring Commercially Available Formulations For Efficient Wound Healing: A Review. Int. J. Environ. Sci..

[147] Pass A., Bialonczyk D., Chiquette E., Goldman J.D. (2021). Oral Superabsorbent Hydrogel (Plenity) for Weight Management. Ann. Pharmacother..

[148] Nuutila K., Laukkanen A., Lindford A., Juteau S., Nuopponen M., Vuola J., Kankuri E. (2017). Inhibition of skin wound contraction by nanofibrillar cellulose hydrogel. Plast. Reconstr. Surg..

[149] Williams C. (1994). Intrasite Gel: A hydrogel dressing. Br. J. Nurs..

[150] Barnea Y., Weiss J., Gur E. (2010). A review of the applications of the hydrofiber dressing with silver (Aquacel Ag) in wound care. Ther. Clin. Risk Manag..

[151] Shiffman M.A. (2018). Introduction to wound dressings. Chronic Wounds, Wound Dressings and Wound Healing; Recent Clinical Techniques, Results, and Research in Wounds.

